# A replication study confirms the association of GWAS-identified SNPs at *MICB* and *PLCE1* in Thai patients with dengue shock syndrome

**DOI:** 10.1186/1471-2350-15-58

**Published:** 2014-05-17

**Authors:** Tran Ngoc Dang, Izumi Naka, Areerat Sa-Ngasang, Surapee Anantapreecha, Sumalee Chanama, Nuanjun Wichukchinda, Pathom Sawanpanyalert, Jintana Patarapotikul, Naoyuki Tsuchiya, Jun Ohashi

**Affiliations:** 1 Faculty of Medicine, University of Tsukuba, Tsukuba, Japan; 2 Department of Medical Sciences, Ministry of Public Health, Nonthaburi, Thailand; 3 Food and drug Administration, Ministry of Public Heath, Nonthaburi, Thailand; 4 Department of Microbiology and Immunology, Faculty of Tropical Medicine, Mahidol University, Bangkok, Thailand

**Keywords:** Association, Dengue shock syndrome (DSS), Expression, MICB, PLCE1, Polymorphism

## Abstract

**Background:**

Dengue shock syndrome (DSS), a severe life-threatening form of dengue infection, mostly occurs in children. A recent genome wide association study (GWAS) identified two SNPs, rs3132468 of major histocompatibility complex class I polypeptide-related sequence B (*MICB*) and rs3765524 of phospholipase C, epsilon 1 (*PLCE1*), associated with DSS in Vietnamese children. In this study, to examine whether an identical association is found in a different population, the association of these two SNPs with DSS was assessed in Thai children with dengue.

**Methods:**

The rs3132468 and rs3765524 SNPs were genotyped in 917 Thai children with dengue: 76 patients with DSS and 841 patients with non-DSS. The allele frequencies were compared between DSS and non-DSS groups by one-sided Fisher’s exact test. The association of rs3132468 and rs3765524 with the mRNA expression levels of *MICB* and *PLCE1* were assessed in EBV-transformed lymphoblastoid cell lines.

**Results:**

The reported DSS-risk alleles were significantly associated with DSS in Thai patients with dengue (one-sided *P* = 0.0213 and odds ratio [OR] = 1.58 for rs3132468-C and one-sided *P* = 0.0252 and OR = 1.49 for rs3765524-C). The rs3132468-C allele showed a significant association with lower mRNA level of *MICB* (*P* = 0.0267), whereas the rs3765524-C allele did not. These results imply that the MICB molecule may play an important role in the prevention of DSS in dengue infection.

**Conclusions:**

Together with previous association studies, we conclude that rs3132468-C at *MICB* and rs3765524-C at *PLCE1* confer risk of DSS in Southeast Asians.

## Background

Dengue is a mosquito-borne disease caused by the dengue virus, a small single-stranded RNA virus comprising four distinct serotypes. Approximately two fifths of the world’s populations (2.5 billion people) are at risk of dengue, and Southeast Asia is one of the most seriously affected regions. The most severe form of dengue is dengue shock syndrome (DSS), a leading cause of death resulting from dengue infection [1].

The clinical outcome of DSS is affected by a number of factors [2,3]. Over the last decade, great efforts have been made to elucidate host genetic factors (i.e., polymorphisms) that are involved in the severity of dengue [4,5]. Recently, a genome wide association study (GWAS) identified two loci, MHC class I polypeptide-related sequence B (*MICB*) and phospholipase C, epsilon 1 (*PLCE1*), associated with DSS in Vietnamese population [6]. Although GWAS provides strong evidence of association, replication studies in different populations or ethnic groups are required to examine whether the same association is commonly found in human populations regardless of the difference in environmental and genetic factors. Furthermore, in the case of infectious diseases, such as dengue, differences in the genetic background of infectious organisms may cause population-specific association. In the present study, in Thai patients with dengue, we investigated the association of two SNPs, rs3132468 and rs3765524, with DSS previously found in Vietnamese patients [6].

## Methods

### Subjects

A total of 917 patients with dengue, treated at Ratchaburi Hospital and Lampang Hospital, Thailand during 1999 to 2004, were examined in this study. All patients were ≤15 years old at diagnosis. The dengue virus infection was confirmed in these patients by dengue IgM/IgG capture ELISA, RT-PCR, and/or dengue virus isolation at the Arbovirus Laboratory, National Institute of Health, Department of Medical Sciences, Ministry of Public Health, Thailand. The patients were classified into three groups: dengue fever (DF), dengue hemorrhagic fever (DHF) and DSS according to the WHO 1997 criteria [7]. DF is defined as an acute febrile stage with various nonspecific symptoms, such as headache, retro-orbital pain, myalgia, arthralgia, rash, hemorrhagic manifestations, leukopenia, and joint and muscular pain. DHF is diagnosed when the patient has additionally all the following symptoms: bleeding (hemorrhagic tendencies), plasma leakage (≥20% change in hematocrit or signs of pleural effusion, ascites, and hypoproteinemia), and thrombocytopenia (platelet count ≤100,000/mm^3^). DSS, a severe form of dengue infection, is defined as DHF with tachycardia (>100/min), narrow pulse pressure (≤20 mmHg), or hypotension (systolic blood pressure <90 mmHg) with cold clammy skin and restlessness.

This study was approved by the Institutional Review Board for Research on Human Subjects, the Department of Medical Science, Ministry of Public Health, Thailand; the Institutional Review Board of the Faculty of Tropical Medicine, Mahidol University, Thailand; and the Research Ethics Committee of the Faculty of Medicine, University of Tsukuba, Japan. Unlinked anonymous blood samples were obtained from the unidentifiable leftover blood of laboratory diagnosis of dengue infection. According to the guideline of Thailand [8], no specific consent forms were required from the patients in this case, since the Ethics Committee approved.

### Genotyping

Genomic DNA was extracted from peripheral blood leukocytes using a QIAamp Blood Kit (Qiagen, Hilden, Germany). Two SNPs, rs3132468 at *MICB* and rs3765524 at *PLCE1*, were genotyped by TaqMan® SNP Genotyping Assay (Applied Biosystems, Foster City, CA, USA).

### mRNA expression data

Normalized mRNA data from Epstein–Barr virus-transformed lymphoblastoid cell lines derived from 45 JPT and 45 CHB HapMap subjects were obtained from the database of the Gene Expression Variation (GENEVAR) project (http://www.sanger.ac.uk/resources/software/genevar/) [9,10]. The mRNA expression data detected by GI_26787987-S probe for *MICB* and GI_19923454-S for *PLCE1* were used in this study. The genotype data of rs3132468 and rs3765524 in HapMap JPT + CHB individuals were obtained from the HapMap database [11,12].

### Statistical analyses

To assess the association of rs3132468 and rs3765524 with DSS, the genotype and allele frequencies were compared by Fisher’s exact test between DSS and non-DSS (DF + DHF). A logistic regression analysis adjusted for age, sex, and geographic location (i.e., hospital) was further performed to assess the association of each SNP with DSS. In a logistic regression analysis, the number of DSS-risk alleles (i.e., 0, 1, or 2) was used as an independent variable. Because the DSS-risk alleles (rs3132468-C and rs3765524-C) were previously identified [6], one-sided *P*-values were calculated. The associations of *MICB* and *PLCE1* SNPs with mRNA expression levels of *MICB* and *PLCE1* gene were evaluated, respectively, using a simple regression analysis with the number of DSS-risk allele as an independent variable. The significance level of a statistical test was set at 0.05 (one-sided *P*-value <0.05 and two-sided *P*-value <0.05 were considered to be statistically significant in association tests of DSS and mRNA expression level, respectively). To search for functional SNPs being in linkage disequilibrium (LD) with rs3132468 (*r*^2^ ≥ 0.8) in HapMap JPT and CHB populations [11,12], an *in silico* tool [13] was used. ESEfinder (http://rulai.cshl.edu/cgi-bin/tools/ESE3/esefinder.cgi?process=home) was used to assess whether SNPs are located in exonic splicing enhancers (ESEs). ESEs are binding sites for Ser/Arg-rich proteins (SR proteins) that have multiple functions in the pre-mRNA splicing process.

## Results

### Patients with dengue

A total of 917 laboratory-confirmed patients with dengue aged ≤15 years old were investigated in this study. Patients were recruited from Ratchaburi Province and Lampang Province, Thailand. The patients with dengue were classified into three groups: 409 patients with DF, 432 patients with DHF, and 76 patients with DSS, according to WHO 1997 criteria (Table 1). In this study, patients with DF and DHF were regarded as patients with non-DSS.

**Table 1:** Subjects investigated in this study

**Characteristics**	**Dengue patients**		
	**DF (n = 409)**	**DHF (n = 432)**	**DSS (n = 76)**
Age (years)^a^	9 (1, 15)	10 (0, 15)	9 (3, 15)
Sex:			
Male	213	235	41
Female	196	197	35
Region:			
Ratchaburi	137	322	52
Lampang	172	110	24
Serotype:			
DEN-1	153	136	15
DEN-2	109	158	39
DEN-3	92	73	6
DEN-4	54	64	16
DEN-3 + DEN-4	0	1	0

^a^Median (minimum, maximum).

### Association test

The allele frequencies of rs3132468-C at *MICB* and rs3765524-C at *PLCE1* were compared between DSS and non-DSS groups (Table 2). Because the significant associations of rs3132468-C and rs3765524-C with risk for DSS have been reported in Vietnamese children, the alternative hypothesis here should be that the allele frequencies of rs3132468-C at *MICB* and rs3765524-C at *PLCE1* were increased in patients with DSS compared with patients with non-DSS. In other words, the one-sided test should be conducted to confirm the previous finding. The one-sided Fisher’s exact test showed that both reported DSS-risk alleles were associated with risk for DSS in Thai children (one-sided *P* = 0.0213 and per allele odds ratio [OR] = 1.58 for rs3132468-C; one-sided *P* = 0.0252 and per allele OR = 1.49 for rs3765524-C).

**Table 2:** **Allelic association test for *MICB *** **rs3132468 and *PLCE1 *** **rs3765524**

**Allele (gene)**	**Dengue patients**		**One-sided *P*-value**	**OR**	**95% CI**
	**DSS**	**non-DSS**			
rs3132468-C (*MICB*)	0.217	0.149	0.0213	1.58	1.02-2.40
rs3765524-C (*PLCE1*)	0.763	0.684	0.0252	1.49	1.00-2.26

*P*-value was calculated by using Fisher’s exact test.

To consider the effects of age, sex, and hospital (i.e., geographic location of subjects) on the association, a logistic regression analysis adjusted for these independent variables was further performed. The logistic regression analysis confirmed the significant association of rs3132468-C at *MICB* and rs3765524-C at *PLCE1* with DSS in Thai patients with dengue (one-sided *P* = 0.0154, per allele OR =1.57, two-sided 95% CI: 1.11–2.22 for rs3132468-C; one-sided *P* = 0.0226, OR = 1.48, two-sided 95% CI: 1.07–2.04 for rs3765524-C). The independent variables, age, sex, and geographic location, showed no significant association with DSS. The results from logistic regression analysis were consistent with those from the Fisher’s exact test.

### Association of DSS-risk allele with mRNA expression level

**Figure 1:**
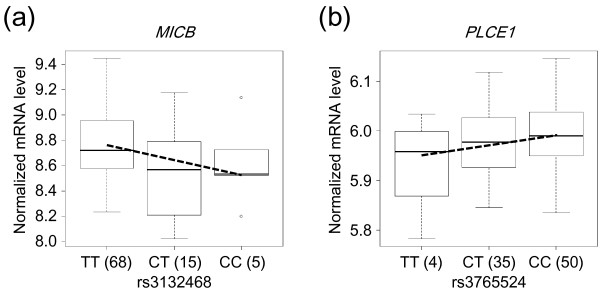
**mRNA expression level and SNP genotype. (a)** mRNA expression level of *MICB* in each genotype of rs3132468. The rs3132468–C allele was associated with lower mRNA expression level of *MICB* (*P* = 0.0267 and partial regression coefficient = −0.126). **(b)** mRNA expression level of *PLCE1* in each genotype of rs3765524. The rs3765524–C allele was not associated with mRNA expression level of *PLCE1* (*P* = 0.0863 and partial regression coefficient = 0.0209). A regression line is represented by a dashed line. The number of samples for each genotype is presented in parentheses.

To examine the functional significance of rs3132468 at *MICB* and rs3765524 at *PLCE1*, the association between the DSS-risk allele and the mRNA expression level was evaluated. The mRNA expression levels of *MICB* and *PLCE1* in EBV-transformed lymphoblastoid cell lines from the HapMap JPT and CHB subjects are shown in Figure 1. The copy number of rs3132468-C in an individual (i.e., 0, 1, or 2) was inversely associated with the level of mRNA expression of *MICB* (*P* = 0.0267 and partial regression coefficient = −0.126), whereas no significant association was observed between rs3765524-C and mRNA level of *PLCE1* (*P* = 0.0863 and partial regression coefficient = 0.0209).

### *In silico* analysis

The rs3132468 SNP is located in intron 6 of *MICB*. Although rs3132468 was statistically associated with risk for DSS, a SNP primarily associated with DSS may not be rs3132468. Therefore, we used an *in silico* tool [13] to search for functional SNPs being in LD with rs3132468. Seventeen SNPs were discovered to be in LD (*r*^2^ ≥ 0.8) with rs3132468 in HapMap JPT population (data not shown). One of these 17, rs3828916, in exon 2 of *MICB*, was predicted by ESEfinder (http://exon.cshl.edu/ESE/) to be located in ESEs that are binding sites for SR proteins, SF2/ASF and SRp40. Because both alleles (i.e., rs3828916-C and rs3828916-G) demonstrated scores larger than the default threshold values (i.e., 1.956 for SF2/ASF and 2.670 for SRp40), it is unclear whether rs3828916 has functional significance. At the *PLCE1* locus, a total of 28 SNPs were in LD (*r*^2^ ≥ 0.8) with rs3765524 in HapMap JPT + CHB populations. Of these 28 SNPs, rs753724, rs2274223, and rs11187870 were predicted to have a functional significance in the *in silico* analysis using the SNPinfo Web Server [13]. The rs753724 SNP located in the upstream region of *PLCE1* was predicted to be located in the transcription factor binding sites. The rs2274223 is a nonsynonymous SNP, which may affect the PLCE1 protein function through amino acid alteration. The rs11187870 SNP was predicted to be located in microRNA-binding sites. Since the above mentioned SNPs may be primary ones with functional significance, we should not conclude that *MICB* rs3132468 and *PLCE1* rs3765524 are causative SNPs associated with DSS.

## Discussion

The present study revealed that *MICB* and *PLCE1* SNPs are associated with a progression from less severe symptom of dengue to DSS in Thai children with dengue. The roles of MICB and PLCE1 proteins in the pathogenesis of DSS remain to be elucidated. MICB is one of stress-induced molecules expressed by virus-infected cells. This molecule interacts with and activates the natural killer group 2 member D (NKG2D) receptor on NK cells. The activated NK cells induce the killing of the virus-infected cells. Our study showed that a DSS-risk allele, rs3132468-C of *MICB*, was significantly associated with lower mRNA expression of *MICB*. As suggested by Whitehorn et al. [14], dengue virus-infected cells with lower MICB expression are plausible to escape from the NKG2D-mediated killing by NK cells. Infected cells escaping the host immune response may mediate the pathogenesis of DSS because high dengue viremia titer is associated with increased disease severity [15]. This requires to be studied in future by the evaluation of the expression levels of MICB and the viremia titer in patients with dengue.

In this study, non-DSS group, compared with DSS group, consisted of patients with DF and DHF. When patients with DSS were compared only with patients with DF, the allele frequencies of rs3132468-C at *MICB* and rs3765524-C at *PLCE1*were significantly higher in DSS group than in DF group (one-sided *P* = 0.0119 for rs3132468 at *MICB* and one-sided *P* = 0.0223 for rs3765524 at *PLCE1*). When patients with DSS were compared only with patients with DHF, the difference in allele frequency of rs3765524-C at *PLCE1* between DSS and DHF groups was statistically significant (one-sided *P* = 0.0399), and the difference in allele frequency of rs3132468-C at *MICB* was marginally significant (one-sided *P* = 0.0521). In contrast, no significant difference in allele frequencies of rs3132468-C and rs3765524-C was observed between DHF and DF groupss (one-sided *P* = 0.1454 for rs3132468 at *MICB* and one-sided *P* = 0.3155 for rs3765524 at *PLCE1*). These results suggest that rs3132468-C and rs3765524-C are not associated with risk for DHF except for DSS in Thai dengue patients.

Previous association studies using cord blood controls suggest that *MICB* and *PLCE1* SNPs are associated with not only DSS [6] but also less severe form of dengue [14]. Our study design allows us to exclude the confounding factors involved in infection with dengue virus and symptomatic dengue infection. However, the limitation is that the possible association of *MICB* and *PLCE1* SNPs with susceptibility to less severe form of dengue cannot be assessed, since only patients with symptomatic dengue infection are available. To examine this possibility in Thai dengue patients, patients with asymptomatic dengue infection remain to be investigated.

## Conclusions

The present study in Thai patients with dengue successfully replicated the associations of *MICB* and *PLCE1* SNPs with DSS that were reported by GWAS in Vietnamese patients [6]. Thus, we conclude that rs3132468-C at *MICB* and rs3765524-C at *PLCE1* are risk factors of DSS in Southeast Asians.

## Competing interests

The authors declare that they have no competing interests.

## Authors’ contributions

TND and JO performed statistical analyses. TND and JO wrote the manuscript. AS and IN extracted DNA. TND performed genotyping. AS, SA, SC, NW, PS and JP collected blood samples and contributed to the acquisition of clinical data. TND, SA, NW, JP, and JO participated in the design and coordination of the study. NT was involved in the interpretation of the data and preparation of the manuscript. All authors read and approved the final manuscript.

## Pre-publication history

The pre-publication history for this paper can be accessed here:

http://www.biomedcentral.com/1471-2350/15/58/prepub
